# Elemental Composition and Strontium Isotopic Ratio Analysis of Industrial Hemp (*Cannabis sativa* L.) for Textile Applications

**DOI:** 10.3390/molecules30234573

**Published:** 2025-11-27

**Authors:** Mirco Rivi, Veronica D’Eusanio, Andrea Marchetti, Emilio Bonfiglioli, Lorenzo Tassi

**Affiliations:** 1 Department of Chemical and Geological Sciences, University of Modena and Reggio Emilia, Via G. Campi 103, 41125 Modena, Italy; mirco.rivi@unimore.it (M.R.); andrea.marchetti@unimore.it (A.M.); lorenzo.tassi@unimore.it (L.T.); 2 National Interuniversity Consortium of Materials Science and Technology (INSTM), 50121 Florence, Italy; 3 Centro Qualità Tessile, Via F. Guicciardini 8/A, 41012 Carpi, Italy

**Keywords:** industrial hemp, elemental composition, strontium isotopic ratio, ^87^Sr/^86^Sr, MC-ICP-MS, sustainability

## Abstract

Industrial hemp (*Cannabis sativa* L.) is increasingly valued as a sustainable raw material for textile applications, yet reliable analytical tools to characterize and trace its origin are still limited. This study presents a pilot investigation on the elemental composition and strontium isotopic ratio (^87^Sr/^86^Sr) of Italian industrial hemp samples, with the aim of evaluating their potential as chemical markers for geographic traceability. Hemp stalks and fibers collected from different Italian regions were finely ground, mineralized using microwave-assisted digestion, and analyzed by atomic absorption spectroscopy (AAS), inductively coupled-plasma mass spectrometry (ICP-MS), and multicollector ICP-MS (MC-ICP-MS). The analytical protocol was validated using certified reference materials, showing recoveries between 95.7% and 102.1%. The measured ^87^Sr/^86^Sr ratios ranged from 0.7085 to 0.7105, with consistent intra-sample reproducibility and values reflecting regional geochemical backgrounds. Elemental profiling revealed marked variability among samples, particularly Sr, Ca, Fe, and trace metals. Principal Component Analysis (PCA) indicated partial clustering according to geographical origin, distinguishing northern from southern Italian samples. Heavy-metal concentrations (Hg, Pb, Cd) were well below international textile safety thresholds, confirming the environmental sustainability of local hemp cultivation.

## 1. Introduction

Hemp cultivation supports the European Green Deal’s goals, offering significant environmental benefits for sustainable agriculture [1]. It sequesters 9–15 tons of CO_2_ per hectare annually, far exceeding forests (2–6 tons) [2]. Its role in crop rotation improves soil health, while its dense canopy prevents erosion and conserves water [3]. Flowering from July to September, hemp boosts biodiversity by providing pollen, bird shelter, and food for wildlife. Additionally, its natural pest resistance minimizes the need for pesticides, herbicides, and fungicides [3].

Italy led global hemp production until the mid-20th century, cultivating over 90,000 hectares by 1940 [4,5]. However, declining demand and prohibition policies nearly eradicated the crop. A revival began in the early 21st century, particularly in textiles and construction, with hemp cultivation reaching 4000 hectares by 2018. The renewed interest in hemp cultivation is largely driven by its versatility, with applications spanning numerous sectors [6]. These include textiles [7,8,9] and packaging [10,11] (e.g., ropes, fabrics, clothing, and sacks), biofuels (e.g., biomass-derived ethanol [12,13]), bio-construction (e.g., hemp–lime composites as thermal insulators [14]), bioplastics [15], paper (a sustainable alternative to wood-based products [16]), cosmetics (e.g., anti-inflammatory and regenerative hemp oil [17]), and antimicrobial applications (e.g., hemp-derived compounds with antibacterial and antifungal properties [18]). Notably, hemp-derived textiles stand out for their sustainability [7,19] as they are natural, biodegradable, eco-friendly, and recyclable, offering a unique combination of breathability, durability, and comfort. These properties place hemp-based fabrics among the most sustainable materials available, rivaling those derived from jute.

To ensure the long-term success of hemp’s resurgence in Italy and safeguard national production, it is crucial to implement reliable characterization systems. Given hemp’s growing economic and environmental significance, robust methods are needed to verify its chemical composition and properties. Characterization could help define quality standards, support the classification of different hemp varieties and uses, and provide a scientific basis for monitoring production practices. In this context, it could also contribute to assessing geographic origin and ensuring greater transparency along the supply chain. Traceability not only help protect Italian hemp from fraud and mislabeling but also reinforce the authenticity and quality of the products in domestic and international markets. One of the most effective approaches involves isotopic and elemental analysis, which allows for a detailed characterization of the hemp plant’s chemical profile. The isotopic composition of certain elements, even when present at trace levels (<1%), can reflect environmental and agronomic conditions experienced during growth. This type of analysis can support the differentiation of samples and the understanding of variability among products and may also provide useful information for developing models of geographic or supply chain traceability when supported by adequate reference data. Radiogenic elements like strontium (Sr), lead (Pb), and neodymium (Nd), derived from rocks and present in soil, often serve as reliable geographic markers [20,21,22]. Among them, the ^87^Sr/^86^Sr ratio is widely used for plant traceability [23]. Strontium has four stable isotopes, with ^87^Sr being radiogenic due to the β-decay of ^87^Rb. Regional Rb/Sr variations create distinctive ^87^Sr abundances. Thanks to its chemical similarity to calcium, Sr integrates into biological systems and their metabolic processes, aiding traceability studies [24]. Isotopic analyses along the supply chain, from soil to the finished product, have demonstrated minimal variation in the ^87^Sr/^86^Sr ratio, suggesting no significant isotopic fractionation during plant metabolism [25]. However, recent advancements have detected slight variations in isotopic ratios, even for elements once considered stable [26]. These differences, though minor, are generally negligible when corrected with standardized methods and are primarily linked to soil properties such as rock type, geological age, and weathering. Developing geographic origin models for plant-based materials requires considering supply chain factors, particularly the bioavailable fraction of elements assimilated by plants. A deeper understanding of these interactions enhances traceability models, supporting hemp’s sustainable development and reinforcing its role in eco-friendly agriculture. The ^87^Sr/^86^Sr isotopic ratio, analysed via Multi Collector-Inductively Coupled-Plasma/Mass Spectrometry (MC-ICP-MS), is a well-established method for determining product origin, as shown in various studies [27]. However, no research has specifically addressed the traceability of textile hemp at the national level. Existing studies have focused on hemp seeds, inflorescences, and stems, using different approaches such as rare earth element (REE) analysis [28] or applications in the food sector [29]. Combining multiple primary indicators, like elemental composition and isotopic ratios, strengthens sample comparison and discrimination, providing a more reliable tool for verifying the origin and authenticity of industrial hemp.

This pilot study on the textile hemp supply chain explores the feasibility of using the strontium isotopic ratio ^87^Sr/^86^Sr and other elemental indicators to characterize textile hemp fibers and gain information about the geographic origin of textile hemp fibers. The main objectives are summarised as follow: (i) developing a sample preparation protocol; (ii) determining the ^87^Sr/^86^Sr ratio and analysing major, minor, and trace elements in hemp samples; (iii) evaluating the link between product and territory.

## 2. Results and Discussion

### 2.1. Fitness for Purpose of the Analytical Method

The fitness for purpose of the analytical methods, in terms of precision, reproducibility and repeatability, were assessed using control samples and certified standards where feasible. This ensured continuous monitoring throughout sample collection, mineralization, instrumental measurement, and container cleaning. This approach allowed for the use of a reduced number of replicates per sample, with the precision determined from the control samples being associated with each measured variable. Analytical method control began with the mineralization process, during which a control sample (sample C) was processed alongside a process blank in every mineralization cycle. The process blank was used to monitor the washing efficiency of the laboratory materials. This procedure was systematically repeated to ensure the complete processing of all available hemp and hemp-based materials. To further verify the operating conditions, a reference material previously analyzed in a separate study [30] and chemically comparable in terms of matrix composition to the current samples was included. This material, labeled Cc (dried vine branch), was specifically used for strontium isotope ratio measurements. The analysis of data obtained from control samples allowed for the assessment of the experimental value distributions for each monitored variable. This step is critical, as it provides an approximate measure of the minimum detectable difference between the analytical data of two samples. To ensure reliability, this difference must be several times greater than the dispersion observed in the corresponding control samples. Consequently, higher precision values (low dispersion of the data) enhance the discriminating power of the variables, improving the ability to distinguish between homologous samples. Moreover, a reference material (NIST SRM 1575a—Pine Needles [31]) was subjected to the full analytical procedure to assess the accuracy of the elemental quantification. The measured concentrations of selected elements were compared to the certified values provided by NIST, and the recovery values (95.7–102.1%) were within the Association of Official Analytical Chemists (AOAC) acceptable ranges, thereby confirming the reliability of the applied method.

#### 2.1.1. AAS

Sr concentration was determined using GFAAS, while Li, Na, Mg, Ca, and K were measured using FAAS. The results are reported in Table S6. Sr concentration is crucial for characterizing hemp samples and supporting the ^87^Sr/^86^Sr isotopic ratio determination. Instrumental mass bias was compensated by maintaining nearly constant Sr concentration in both sample solutions and standards. To achieve an optimal signal-to-noise ratio for all isotopes, the element concentration was adjusted to match the reference concentration, allowing a maximum deviation of ±20%, with a target concentration of 200 μg kg^−1^ of Sr. The “C” hemp sample, the control sample, underwent the complete analytical procedure, including grinding and autoclave mineralization, ensuring the robustness and reproducibility of the method. Lithium (Li) was measured using atomic emission due to its higher sensitivity. Replicates of the control sample (C) showed consistent results, confirming the reliability of the method and equipment. With the exception of some elements, such as Na and Li, the RSD % values agree very well with the expected values from the Horwitz diagram, confirming the good measurement conditions [32].

#### 2.1.2. Determination of Elements Using ICP-MS Technique

Trace and ultratrace element concentrations in hemp samples were determined using inductively coupled-plasma mass spectrometry (ICP-MS) with a triple quadrupole mass analyzer, ensuring high analytical precision in complex matrices. Reliability was verified through multiple processing sessions, using control sample C and process blanks. The quantified isotopes included: ^11^B, ^52^Cr, ^55^Mn, ^56^Fe, ^58^Ni, ^59^Co, ^63^Cu, ^64^Zn, ^85^Rb, ^98^Mo, ^111^Cd, ^120^Sn, ^138^Ba, ^202^Hg, ^205^Tl, ^208^Pb and ^209^Bi. Control sample C exhibited minimal value distribution (Table S6), confirming excellent method repeatability and reproducibility, with relative standard deviations (RSDs) ranging from 2% to 12% for trace and ultratrace elements, respectively.

#### 2.1.3. Determination of ^87^Sr/^86^Sr Ratio

The separation of strontium from interferents is crucial for accurate isotopic ratio determination. Rubidium (Rb) concentrations exceeding 50 μg kg^−1^ in the final sample (with Sr concentration at 200 μg kg^−1^) can introduce errors ten times greater than typical measurement uncertainty [33]. To minimize isobaric interference from ^87^Rb, the separation process must effectively remove this element while ensuring high Sr recovery. During separation, rubidium and other interferents are eliminated in the washing eluate, while Sr is selectively retained and subsequent released. The efficiency of this process is verified by monitoring residual Rb levels and Sr concentrations in the processed solution. For reliable isotopic ratio measurements, Sr recovery must exceed 93–95% to prevent mass-dependent fractionation phenomena. Analytical recovery was assessed by comparing Sr concentrations before and after solid-phase extraction (SPE) on the Sr selective resin. The recovery tests yielded values between 93% and 102%, with an average recovery of (98 ± 6, *n* = 8) %, confirming the method’s suitability for precise isotopic analysis.

The precision of ^87^Sr/^86^Sr ratio determination was assessed by repeated analysis of control samples C and Cc. Considering the greater criticality of the isotope ratio measurement process, a second control sample was introduced. This sample was the subject of numerous determinations within another project [25,30]. The combined evaluation of resin separation, measurement procedures, and mathematical corrections yielded highly precise values. Sample C exhibited an average ^87^Sr/^86^Sr ratio of 0.70877 ± 0.00007 (mean ± 2sd, *n* = 14), with measurement uncertainty consistent with previous studies on similar matrices [30]. For sample Cc, the ratio was 0.70925 ± 0.00002, (mean ± 2sd, *n* = 4), closely matching the value of 0.70927 ± 0.00002, (mean ± 2sd, *n* = 5), obtained in 2012–2013 during the AGER project [34,35]. The difference between the groups was not significant: *t*(6) = −0.905, *p* = 0.400.

The separation method’s accuracy and precision were further validated using the certified isotope standard Sr(CO_3_)_2_, NIST 987 (accepted ratio: 0.71026 ± 0.00002, mean ± 2sd [36]). Measurements of NIST 987 after resin separation and during the bracketing procedure confirming that ^87^Sr/^86^Sr values consistently fell within the accepted confidence interval, demonstrating method reliability and instrumental stability (Figure S3 of Supplementary Materials).

The average experimental value of the ^87^Sr/^86^Sr ratio was found to be 0.71027 ± 0.00002 (mean ± 2sd, *n* = 84). From a statistical point of view, this value is comparable to the certified ratio, confirming the accuracy of the measurement process. Furthermore, the average value obtained in this study for the NIST 987 standard is in perfect agreement with the historical average determined by the laboratory over the period of spectrometer operation (2010–2024), which is ^87^Sr/^86^Sr = 0.71027 ± 0.00008 (mean ± 2sd, *n* = 2669). To compare the means obtained in this study with the historical data, a statistical t-test was conducted. The difference between the groups was not significant: *t*(207) = −0.328, *p* = 0.743.

### 2.2. Characterization of Hemp Samples

#### 2.2.1. Determination of ^87^Sr/^86^Sr Ratio

The analyzed hemp samples, primarily of national origin, were received in various physical forms, including dried plants and fibers, grounded material, and fiber-shive mixtures. To maximize data collection, two “hemp stem” samples were further characterized by dividing the plant into four sections from base to apex (see details in Supplementary Material, Table S1). Since nutrient distribution varies across plant tissues, this study aimed to assess potential differences between plant sections and between fiber (outer part) and shives (inner woody part).

Starting from the base and arriving at the apex, the four sections obtained are: A0_1–A0_4 and A1_1–A1_4 (Supplementary Material Figure S4). The isotopic ratios were consistent across sections, with mean values of ^87^Sr/^86^Sr = 0.70864 ± 0.00005 (u = 2sd) for A0 and ^87^Sr/^86^Sr = 0.70860 ± 0.00001 (u = 2sd) for A1, indicating minimal variation within the sample. Since both plants originated from a single bundle, these results were expected. Notably, the values represent the combined “fiber + shives” matrix, as the analyzed sections were fully ground.

A separate analysis focused solely on the fiber, obtained by crushing dried stems and isolating the external filamentous portion, was performed. The fiber sample (A2) yielded an isotopic ratio of ^87^Sr/^86^Sr = 0.70856 ± 0.00007, statistically comparable to the mean of A0 and A1 (mean ± u; 0.70862 ± 0.00005).

**Figure 1:**
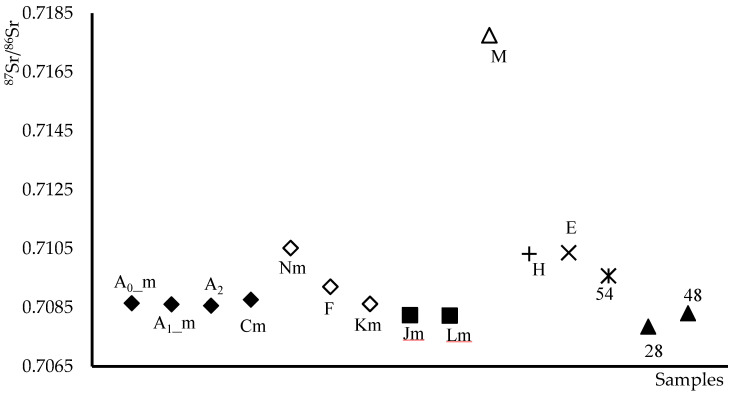
Distribution by geographical macro areas of the ^87^Sr/^86^Sr isotope ratio values of hemp samples. (◆) Carpi(MO); (**◇**) Modena; (**✕**) Udine: (**+**) Brennero; (■) Parma; (▲) Bari, Caserta; (**✴**) Rovigo; (**△**) Brunico. The letter “m” indicates the mean value of the studied samples.

These findings confirm that isotopic ratio measurements can be reliably obtained from both whole plant stems and isolated fiber. The isotopic data for all hemp samples, including those of different origins, are presented in Figure 1. While the sample set is not fully representative of Italian hemp production, the measured values align with expected ranges for their presumed geographical origins, which will be further discussed.

The distribution of samples shown in Figure 1 offers a broader perspective for interpretation. It is immediately evident that most samples exhibit isotopic ratios below 0.71, indicating low radiogenicity. This feature narrows the variability range, complicating the possibility of differentiation of samples based on geographical origin. However, as this is a preliminary study, only a limited number of samples were available for analysis. Expanding the dataset in future investigations may provide additional insights into the potential for geographical traceability.

Additionally, the discriminatory power of the ^87^Sr/^86^Sr ratio is further limited by high intra-site variability: multiple samples originating from the same macro-area display overlapping isotopic signatures. This is the case for samples A_0_m, A_1_m, A_2_, Cm, F, Km, and Nm, all sourced from the area of Carpi (MO) or the wider Modena province. Now, by assuming as accurate the declared geographical origins of the samples, the average ^87^Sr/^86^Sr value for sample Nm (0.71052 ± 0.00008) stands out as notably higher than the mean value for the other samples from the same area (^87^Sr/^86^Sr = 0.70873 ± 0.0004).

If sample N genuinely originates from the Modena area, this would imply that the regional isotopic range extends from 0.7105 to 0.7087. Within such a narrow interval, distinguishing the remaining samples becomes unfeasible, except for samples M. However, the considerable deviation observed for sample N suggests it may not, in fact, be of Modena origin.

In a previous study focused on developing a geographical traceability model for Lambrusco wines from the Modena province [37], a dataset was generated that enabled the construction of a regional ^87^Sr/^86^Sr isotopic map. This map highlights that the area encompassing the hinterland of Modena and the northern plains, zones historically and agriculturally favorable for hemp cultivation, is characterized by isotopic ratio values within the range of 0.7083 < ^87^Sr/^86^Sr < 0.7092.

These values are in line with those obtained for the hemp samples from the Carpi area, as well as samples F and Km from the Modena province, supporting the consistency and reliability of the isotopic data in reflecting the local geochemical background.

#### 2.2.2. Elemental Content in Hemp Samples

The determination of metal content in plant-based materials is often explored as a tool to investigate potential sources of variability, including environmental factors and plant physiology. Although elemental composition can, in some cases, reflect the characteristics of the soil in which a plant grows, establishing geographical traceability requires extensive dataset, controlled sampling strategies and well-defined reference models. Moreover, the transfer of elements from soil to plant, referred to as translocation, is influenced by complex interactions involving soil properties, water availability, elemental bioavailability, pH, temperature and species-specific physiological traits. Once absorbed, the elements may also be redistributed unevenly among plant organs depending on their function, further contributing to intrinsic variability. In light of these considerations, the aim of the present study was not to infer geographical origin, but rather to provide a preliminary description of the variability of major and trace elements in the analysed hemp samples. This characterization represents an initial step toward understanding how elemental profiles may differ between plant tissues and processing fractions, acknowledging the limitations imposed by the sample set and available data.

**Figure 2:**
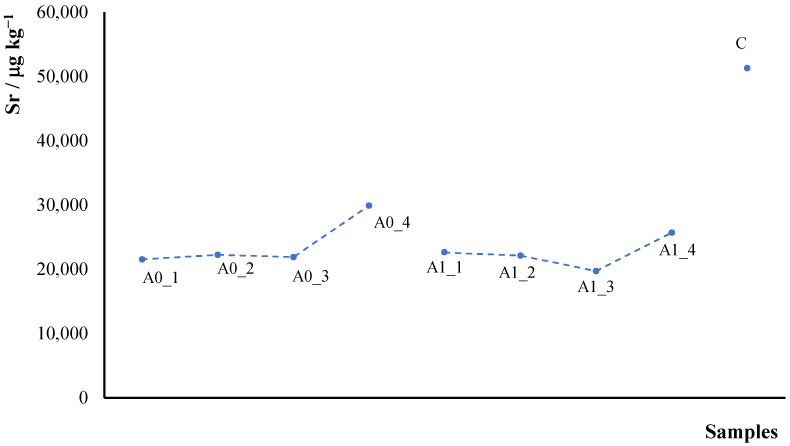
Sr concentration, μg kg^−1^, determined in the hemp samples obtained by cutting the stem of plants, A0_1–4 and A1_1–4, and value measured on the C fiber.

As an illustrative example, Figure 2 presents data on strontium (Sr) concentration in hemp samples derived from sectioned whole plant stems. The concentrations were measured in solutions obtained through mineralization of four distinct segments of the stem.

The resulting data reveal a consistent pattern: Sr concentration remains almost constant from the base toward the ¾ of the length of the plant and increase towards the apical section (from left to right in the graph). Notably, the external fiber portion of the plant exhibits even higher Sr concentrations than the internal segments. This pattern was consistently observed across all elements analyzed in this study. This trend can be explained considering the physiological transport mechanisms in hemp. As reported for other plant species, the upward increase in metal concentration likely reflects the xylem-driven translocation of elements from roots to metabolically active tissues, where nutrient demand is higher [38,39,40]. The apical regions of the stem are closely associated with developing leaves and meristematic tissues, which generally exhibit enhanced accumulation of both essential and non-essential elements due to higher transpiration rates and active metabolism [41]. Previous studies have shown that metals such as Ca, Mg, Fe, and Sr are often more concentrated in leaves than in stems, suggesting that their gradient along the stem mirrors the direction of nutrient flow toward photosynthetically active organs. Moreover, the higher Sr concentration observed in the outer fibers compared to inner tissues may be related to differences in cell wall composition and cation-exchange capacity [42], as the fiber fraction is rich in pectins and hemicelluloses capable of binding divalent cations such as Sr^2+^ [43].

**Figure 3:**
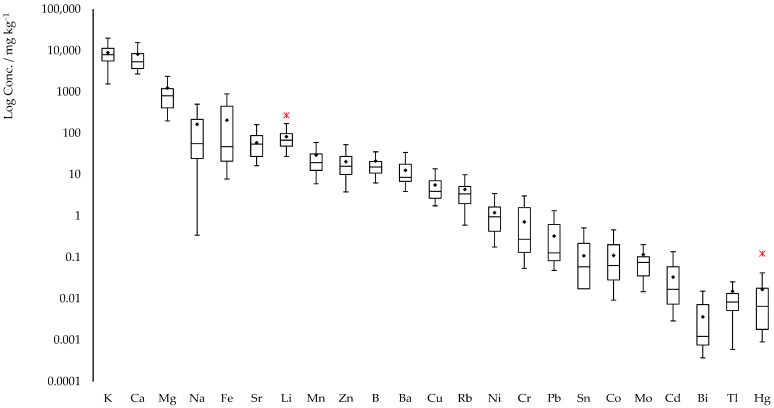
Box and whiskers plots distribution of concentrations, mg kg^−1^, of the elements determined in the hemp samples. Data are reported in Log scale. Outliers are marked by an asterisk (*).

To visualize these trends, Figure 3 displays box plot distributions of the elemental concentration data, reported on a logarithmic determined in the hemp samples. These visualizations further support the relevance of element content as a discriminating factor in geographic traceability efforts.

The elemental composition of the analyzed hemp samples displays a broad range of variability. This diversity complicates the interpretation of data based solely on sample provenance, particularly given the high number of both samples and elements considered. Regarding the concentrations of elements obtained in this study, the values align well with those reported in the literature for hemp plants [44,45,46,47,48] and seeds [49]. For example, the Cr concentration measured in our samples (719 μg kg^−1^) is comparable with literature values, which range from 340 to 7890 μg kg^−1^ in Turkish samples [49], up to 17,410 μg kg^−1^ in Nigerian hemp [45]. Similar agreement was found for Ni, with our study reporting 1206 μg kg^−1^, in line with literature values ranging from 550–1660 μg kg^−1^ in Turkish samples [49], and even higher values observed in Nigerian (6800 μg kg^−1^) and Greek hemp (2300–48,960 μg kg^−1^) [45,48]. The data from the Greek study [48] are particularly noteworthy, as they highlight hemp’s remarkable phytoextraction capacity in contaminated soils. In Greek samples, high levels of several elements were observed, including Mn (76,900–518,800 μg kg^−1^), Fe (135,600–1,338,400 μg kg^−1^), Cu (8200–64,200 μg kg^−1^), Zn (23,100–158,000 μg kg^−1^), Sr (35,948–1,032,403 μg kg^−1^), Cd (7–431 μg kg^−1^), Sn (14–181 μg kg^−1^), Hg (6–107 μg kg^−1^), and Pb (95–1752 μg kg^−1^). These values, among the highest reported, support the use of hemp for phytoremediation purposes and underscore the need for site-specific monitoring when hemp is cultivated on contaminated soils. The results of this study also show that the analyzed industrial hemp samples generally contain significantly lower concentrations of heavy metals compared to those from more heavily contaminated regions. Given the well-documented toxicity of heavy metals to various organisms, elevated levels in plant materials may pose serious concerns when hemp or its derivatives are utilized in industrial valorization processes, such as fiber production, building material manufacturing, or energy recovery.

Despite these concerns, no specific legal limits currently exist at the European or Italian level regarding metal concentrations in industrial hemp for textile applications. However, voluntary standards and certifications, such as Standard 100 by OEKO-TEX^®^ [50] available online: https://www.oeko-tex.com/en/our-standards/oeko-tex-standard-100/ accessed on 10 May 2025), establish stringent criteria for the presence of harmful substances in textile products. These standards play a crucial role in ensuring the safety and compliance of textiles intended for human use, providing an additional layer of quality control in the absence of binding regulatory thresholds and the samples analyzed in this study fall within the limits established by the standard.

Regarding regional variability, only limited trends could be identified, likely due to the restricted number of samples analyzed in this preliminary study. However, a discussion of potential regional trends in elemental composition is presented in the following section (Section 2.2.3).

#### 2.2.3. PCA

**Figure 4:**
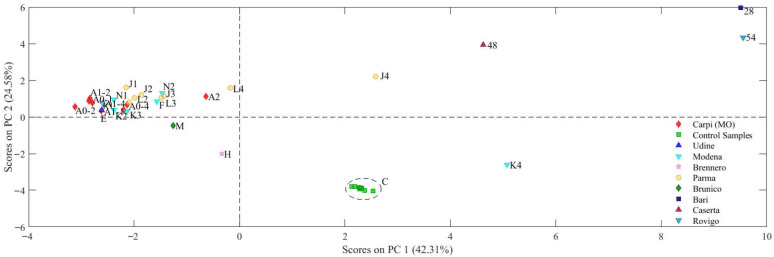
Score plot (PC1 vs. PC2). Carpi (MO), Udine, Modena, Brennero, Parma, Brunico, and Rovigo samples are from northern Italy, while Bari and Caserta samples are from the south.

**Figure 5:**
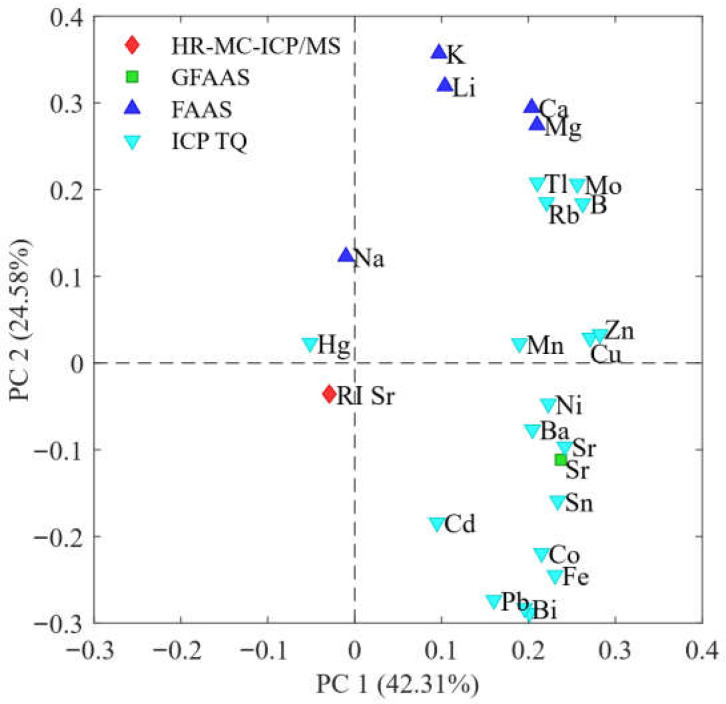
Loadings plot (PC1 vs. PC2).

To compare and comment on the different behaviors of the samples in relation to the monitored variables, a Principal Component Analysis was performed. This technique allows us to identify and visualize any patterns or similarities between the samples based on the variables investigated. The PCA applied to the experimental dataset produced a model consisting of four PCs with an explained total variance equal to 80%. In particular, Figure 4 and Figure 5 report the Factor Scores and Loadings of the first two components PC1 vs. PC2. In particular, the first two components of the model explain a variance of more than 60% of the total. This indicates that a significant part of the variability of the data is captured by these two components. In the score plot, most of the samples appear grouped in a single cluster, suggesting a certain homogeneity between these samples.

Analyzing the loadings graph (Figure 4), it is observed that Hg and ^87^Sr/^86^Sr isotope ratio contribute significantly to the distribution of the samples along the first principal component (PC1), providing a clear distinction between the samples along this dimension.

As for the second principal component (PC2), the elements that mostly influence the distribution of the samples are the contents of Li, Bi, Pb and Ca. This suggests that, in addition to the main elements highlighted for PC1, the concentration of Zn, Cd and Sr plays an important role in differentiating the samples along PC2 where samples with a higher content of Li, K, Mg and Ca position the samples at positive values while the other measured concentrations such as Bi, Pb, Fe and Co influence PC2 bringing the samples to the negative hemisphere.

Indicatively, it can be observed that the samples from Carpi, Modena and Parma are mainly arranged in the negative part of the first principal component (PC1). This behavior is due to the fact that these samples present lower values for most of the parameters considered, except for the concentration of Hg and the RI Sr. These two parameters, in fact, are more represented in these samples than in the others, as highlighted by the loadings graph (Figure 4).

On the contrary, the samples that are arranged towards positive values of PC1 mainly come from Southern Italy. This suggests that these samples have a distinct chemical profile compared to the northern ones, characterized by a higher concentration of the parameters that can be seen in the loadings graph.

As for the control samples (C), they are located in the quadrant characterized by positive values of PC1 and negative values of PC2. This arrangement is due to the concentrations of certain elements that, as illustrated in the loadings graph, appear to have a strong negative influence along the PC2 axis. This suggests that the control samples have a distinct chemical composition, influenced by a different distribution of some elements compared to the other samples analyzed, along the axis of the second principal component (PC2).

This approach allows better observation of variations between samples with respect to this specific isotopic parameter.

In the score plot graph, it is clearly noted that samples from southern Italy present lower isotopic ratio values, positioning themselves in the quadrants with negative PC1 values. On the contrary, samples from northern Italy show a higher ^87^Sr/^86^Sr isotopic ratio, with a distribution in the quadrants characterized by positive values for PC1. This suggests a geographical distinction in the isotopic distribution between the different Italian regions.

Unlike the samples from Carpi, Modena and Parma which are positioned in neighboring areas and consequently produce a cluster of samples, the samples from Southern Italy have a much wider arrangement in the graph since they cover a larger geographical area and consequently present different concentrations.

In summary, the arrangement of the samples along the main components reflects regional and compositional differences, highlighting a clear separation between the samples from Northern and Southern Italy, as well as a distinctive characterization of the control samples.

## 3. Materials and Methods

### 3.1. Reagents and Materials

Ultrapure 65% HNO_3_ was obtained from analytical grade HNO_3_ (Carlo Erba, Milan, Italy) using a SAVILLEX DST 1000 sub-boiling system (Savillx Corp., Eden Prairie, MN, USA). High-purity water (ASTM Type I) was obtained by a Millipore IQ 7000 combined with an IQ Element system (Millipore, Milan, Italy). The ICP-multielement solutions IV-ICP-MS-71A and IV-ICP-MS-71B, used for element concentration determination, was obtained from Inorganic Ventures (Christiansburg, VA, USA). SrCO_3_, NIST SRM 987 (NIST, Gaithersburg, MD, USA), with a certified ^87^Sr/^86^Sr value of 0.71034 ± 0.00026 (the uncertainty “u” is expressed as twice the standard deviation, u = 2sd) [51], and a generally accepted ratio of 0.710263 ± 0.000016 (u = 2sd) [36] was used for the bracketing procedure and data precision evaluation. The Eichrom SR-B100-S (50–100 μm) Sr resin, used for Sr/Rb separation, was purchased from Eichrom Technologies Inc. (Lisle, IL, USA). All Perfluoroalkoxy (PFA) bottles, tubes and vessels used for solution and sample preparation were first washed with heated 10% sub-boiled HNO_3_ and then rinsed several times with high-purity water. Standard solutions and sample preparations were performed gravimetrically using a Mettler AE200 analytical balance (Mettler Toledo S.p.A, Milan, Italy) with a 0.0001 g sensitivity. All standards and samples were processed in a cleanroom laboratory equipped with HEPA filters (Faster S.r.L., Milano, Italy) to prevent contamination.

### 3.2. Sampling

The industrial hemp samples were obtained from Centro Qualità Tessile (Carpi, Modena, Italy) and the samples characteristics and the sampling sites are shown in Supplementary Materials (Table S1 and Figure S1). To investigate the distribution of elements within different sections of the hemp plant, the stalks were divided into four distinct portions. The basal section, closest to the root, was labelled as portion 1, while the apical section, nearest the tip, was labelled as portion 4. Additionally, for further analysis, samples were taken by separating the outer layer from the central hurd to examine potential variations in isotopic ratios and other elemental compositions that could be associated with the sample type. For samples consisting of fiber or mixed fiber stalks, no segmentation or separation was conducted.

### 3.3. Grinding

The samples were then shredded and ground to achieve a homogeneous product. Grinding was performed by using a centrifugal mill (Fritsch model Pulverisette 14, Laborgeraetebau, Neuenrade, Germany), equipped with a 12-blade rotor and a 500 μm trapezoidal mesh sieve, both made of titanium. A stainless-steel collection container lined with Teflon was used to collect the ground material. The mill was operated at a speed of 16,000 rpm to prevent overheating of the material during the grinding process.

### 3.4. Sample Mineralization

For analytical preparation, wet digestion was employed to obtain solutions suitable for the techniques utilized in this study. The digestion process was conducted using a microwave-assisted autoclave operating on the Single Reaction Chamber (SRC) principle (Ultrawave, Milestone, Bergamo, Italy). Approximately 0.5 g of each finely ground sample was accurately weighed, followed by the addition of an oxidizing mixture comprising 6 mL of concentrated HNO_3_ and 4 mL of H_2_O. The samples were then sealed in the pressurized autoclave, where digestion was performed under controlled conditions: initial N_2_ pressure between 30 and 40 bar. The heating program is reported as Supplementary Material in Table S2 and Figure S2).

### 3.5. Atomic Absorption Spectroscopy (AAS)

This spectroscopy technique was employed for the quantification of Li, Na, K, Ca, and Mg. The measurements were performed using a Varian Spectra AA FS220—HGA100 (Agilent Technologies, Santa Clara, CA, USA) instrument, equipped with a SIPS10 sample introduction and dilution system. Standard solutions were prepared by a serial dilution of a multielement stock solution containing Li (1.053 ppm), Ca (1.001 ppm), K (0.4009 ppm), Mg (0.1937 ppm) and Na (0.3232 ppm). Calibration was periodically performed after 10 sample reading to compensate for instrumental drift.

The concentration ranges for the calibration curves were element-specific: Li (0.2–1 ppm), Na (0.06–0.3 ppm), K (0.08–0.4 ppm), Mg (0.04–0.2 ppm), and Ca (0.2–1 ppm). The calibration curves consistently exhibited a coefficient of determination (R^2^) of no less than 0.999, ensuring high reliability. Notably, no deuterium lamp background corrections were applied during the measurements. Additional experimental parameters are summarized in Supplementary Material (Table S3).

Graphite furnace atomic absorption spectrometry (GFAAS) was employed for the quantification of strontium in the samples. Calibration standards were prepared by appropriate dilutions of a stock solution containing 14.78 ppb of Sr, covering a concentration range of 4–13 ppb. The resulting calibration curves consistently exhibited coefficient of determination (R^2^) values exceeding 0.9999. To ensure accurate measurements, the samples were appropriately diluted prior to analysis. Measurements were conducted using the temperature program detailed in Supplementary Material (Table S4).

### 3.6. ICP-MS Mass Spectrometry

An initial survey was conducted to determine the target elements for measurement, which included ^11^B, ^52^Cr, ^55^Mn, ^56^Fe, ^59^Co, ^60^Ni, ^63^Cu, ^68^Zn, ^85^Rb, ^88^Sr, ^98^Mo, ^111^Cd, ^118^Sn, ^137^Ba, ^202^Hg, ^205^Tl, ^208^Pb, and ^209^Bi (Supplementary Material Table S5) using a iCAP TQ ICP-MS system (Thermo Scientific, Waltham, MA, USA). Calibration curves for each element were generated using multistandard solutions and following appropriate dilutions. Internal standards (^45^Sc, ^89^Y and ^115^In) were employed to enhance measurement accuracy. The analysis was performed with a triple quadrupole system using oxygen (O_2_) as a reaction gas and utilizing kinetic energy discrimination (KED) to improve specificity and precision. Procedural blanks were on average <1.3 ppt for the investigated elements.

### 3.7. The MC-ICP-MS Mass Spectrometer

For the determination of the ^87^Sr/^86^Sr isotopic ratio, mineralized solutions were prepared through Sr/Rb separation using Eichrom selective resin. Rubidium (^87^Rb) present in the samples was identified as the primary interferent in measuring the ^87^Sr/^86^Sr ratio via MC-ICP-MS. The separation procedure has been reported and discussed in a previous work [21] and the main steps are herein briefly reported: (i) 1 mL volume of resin was loaded into the solid-phase extraction (SPE) column. The resin was washed with 2 mL of ultra-pure water and activated using 5.5 mL of 8 M HNO_3_; (ii) the sample was loaded onto the column, and interferents, including rubidium, were eluted with 3.5 mL of 8 M HNO_3_. The strontium fraction was subsequently collected using 9.5 mL of ultra-pure water; (iii) to adjust the solutions to a final nitric acid concentration of approximately 4%, 1 mL of 65% HNO_3_ was added prior to analysis.

The strontium concentration in the samples was determined both before and after the separation process using graphite furnace atomic absorption spectrometry (GFAAS).

Strontium isotopic measurements were performed using a multicollector inductively coupled-plasma mass spectrometer (MC-ICP-MS) with a Forward Nier–Johnson geometry (Neptune, ThermoFinnigan, Bremen, Germany). Information regarding ICP-MS operating parameters for the ^87^Sr/^86^Sr ratio measurements are reported in Table S7. The instrument featured a multicollector configuration with nine Faraday cups: eight movable and one fixed central collector. Ion lens settings were optimized daily to achieve maximum sensitivity and a well-defined flat-top peak shape for precise measurements. Instrumental drift was corrected using a bracketing sequence in the order [blank/standard/blank/sample/blank]. The average intensity of the blank measurements (4% HNO_3_) was calculated and subtracted from the intensity measurements of the corresponding standards and samples. Blank levels contributed, on average, 0.2 µg kg^−1^ to the measured ^88^Sr concentration. The strontium isotope ratios of the standards and samples were then calculated following the mathematical procedures outlined in a previous study [25]. These calculations included an internal correction for mass discrimination effects induced by the plasma source, employing the normalized ^88^Sr/^86^Sr ratio of 8.3752, as recommended by the IUPAC technical report on isotopic composition [52]. Additionally, mathematical corrections for krypton (^86^Kr) and rubidium (^87^Rb) interferences were performed by applying the ^86^Kr/^83^Kr and ^87^Rb/^85^Rb ratios of 1.50566 and 0.38567, respectively.

All isotopic values obtained for the NIST SRM 987 standard during each measurement session were used to evaluate the instrumental precision/accuracy.

### 3.8. Data Analysis

The mean, standard deviation values and Welch’s *t* Test were calculated using the statistical functions of Microsoft Excel software, ver. 2510 (Excel^®^ for Microsoft Office 365, Microsoft^®^, Redmond, WA, USA). The level of significance was set at *p* < 0.05 to determine whether there were statistical differences among the mean values. PCA was conducted by using Matlab R2023a (The Mathworks Inc., Natick, MA, USA). Prior to PCA, data were normalized by mean-centering and scaling to unit variance in order to remove the effect of different measurement scales.

## 4. Conclusions

This study was conceived as a pilot project to assess the feasibility of supporting industrial hemp-derived product development using raw materials from local and national sources. The main objective was to evaluate the suitability of hemp for integration into the textile supply chain through advanced analytical methodologies. In this regard, a robust sample pre-treatment protocol, specifically adapted for the challenging matrix of industrial hemp, was successfully optimized. This protocol ensured the high purity required for accurate Sr isotopic analysis and achieved complete sample digestion for reliable multi-element profiling.

Using a multi-indicator approach combining isotopic and elemental analyses, variability was observed in the ^87^Sr/^86^Sr ratios among samples collected from different Italian regions. Samples from Carpi, Modena, and Parma showed comparable isotopic signatures, whereas those from southern Italy displayed distinctly lower radiogenic values, indicating the potential of this parameter to differentiate hemp according to geographical origin. Moreover, elemental profiling revealed a vertical distribution of specific metals within the plant, with higher concentrations in the apical sections. Elements of toxicological concerns, such as Hg, Pb, and Cd, were detected at very low levels.

Overall, the combined use of ^87^Sr/^86^Sr isotopic ratio with elemental concentration profiles proved an effective tool for the chemical characterization of industrial hemp. While these tools may support the development of traceability models when adequate reference data are available, their primary value lies in enhancing our understanding of the variability and properties of hemp grown under different environmental and agronomic conditions. However, given the pilot nature of this study and the limited sample size, which does not comprehensively cover the full geographical and agronomic diversity of Italian hemp production, the observed isotopic and elemental pattern should be interpreted as preliminary indications. The results are robust for the regions and cultivation practices directly sampled. Future research must substantially expand the sampling network to include a broader range of geographical areas, soil types, and hemp varieties, and integrate multi-year datasets to account for seasonal and climatic variability.

## Data Availability

Data are available within the article.

## Supplementary Materials

The following supporting information can be downloaded at: https://www.mdpi.com/article/10.3390/molecules30234573/s1, Figure S1: Map of sampling sites; Figure S2: Trend of the temperature (blue line), pressure (green line), and autoclave cylinder temperature (orange line) parameters relating to the mineralization process of the hemp samples; Figure S3: Trend of the isotope ratio values for the NIST 987 standard. The figure shows the generally accepted ratio values of 0.71026 and the associated uncertainty interval ±0.00002; Figure S4: ^87^Sr/^86^Sr isotope ratio values determined on the hemp pieces (black circular indicator) and on the A2 fiber sample (square indicator). The vertical lines denote the uncertainty value, u = 2sd, associated with determinations equal to ±0.00005 for the series and ±0.00007 for the A2 fiber sample; Table S1: Tested samples with their brief description and origin; Table S2: Mineralization ramp of the samples. E, microwave power; t, duration time of the step; T1, internal temperature of the container; T2, external limit temperature of the magnetron; P, maximum pressure of the mineralization chamber; Table S3: Experimental conditions used for the determination of Li, Na, K, Mg, and Ca using the FAAS technique; Table S4: Heating program for the determination of Sr concentration using the GFAAS technique. The asterisked steps represent the signal reading stages; Table S5: Mass/charge ratio value measured for each element examined; Table S6: Values of the elements measured for the control sample C; Table S7: Multi collector ICP-MS operating parameters for the ^87^Sr/^86^Sr ratio measurements.
