# Supplementary material for: Elemental Composition and Strontium Isotopic Ratio Analysis of Industrial Hemp (Cannabis sativa L.) for Textile Applications
Source: Molecules. 2025 Nov 27;30(23):4573. doi: 10.3390/molecules30234573 (PMC12692789; doi:10.3390/molecules30234573)
Supplement: Supplementary file 1 [file molecules-30-04573-s001.zip › molecules-3956402-supplementary.pdf]

Supplementary materials

Table S1. Tested samples with their brief description and origin.

Sample	Characteristics	Source	GPS
**A**	Hemp stem, length: 2 m	Carpi	44.706046, 10.847027
**A_{0-1}**	First quarter, basal of the hemp stem, length: 50 cm	Carpi	44.706046, 10.847027
**A_{0-2}**	Second quarter of the hemp stem, length: 50 cm	Carpi	44.706046, 10.847027
**A_{0-3}**	Third quarter of the hemp stem, length: 50 cm	Carpi	44.706046, 10.847027
**A_{0-4}**	Apical section of the hemp stem, length: 50 cm	Carpi	44.706046, 10.847027
**A_{1-1}**	First quarter, basal of the hemp stem, length: 50 cm	Carpi	44.706046, 10.847027
**A_{1-2}**	Second quarter of the hemp stem, length: 50 cm	Carpi	44.706046, 10.847027
**A_{1-3}**	Third quarter of the hemp stem, length: 50 cm	Carpi	44.706046, 10.847027
**A_{1-4}**	Apical section of the hemp stem, length: 50 cm	Carpi	44.706046, 10.847027
**A_2**	External part of the Carpi stem obtained by separation by the central part	Carpi	44.706046, 10.847027
**C**	Fibrous part of the Carpi stem	Carpi	44.706046, 10.847027
**E**	Fedora variety hemp stem mixed with fiber, harvested in 2022	Udine	46.027855, 13.282296
**F**	Hemp stem mixed with fiber	Modena	44.623222, 10.997917
**H**	Hemp stem, harvested in the 2022	Brennero	46.996725, 11.500710
**J_1**	Hemp stem, harvested in 2020, length: 2 m; basal section of the stem, length 50 cm	Parma	44.837437, 10.434464
**J_2**	Hemp stem approximately 2 meters high harvested in 2020. Central section closest to the basal part of the stem, length: 50 cm	Parma	44.837437, 10.434464
**J_3**	Hemp stem approximately 2 meters high harvested in 2020. Central section furthest from the basal part of the stem, length: 50 cm	Parma	44.837437, 10.434464
**J_4**	Hemp stem approximately 2 meters high harvested in 2020. Central section furthest from the basal part of the stem, length: 50 cm	Parma	44.837437, 10.434464
**K_1**	Hemp stem approximately 2 meters high harvested in 2023. Basal section of the stem, length: 50 cm	Modena	44.592862, 10.994557
**K_2**	Hemp stem approximately 2 meters high harvested in 2023. Central section closest to the basal part of the stem, length: 50 cm	Modena	44.592862, 10.994557
**K_3**	Hemp stem approximately 2 meters high harvested in 2023. Central section furthest from the basal part of the stem, length: 50 cm	Modena	44.592862, 10.994557

**K_4**	Hemp stem approximately 2 meters high harvested in 2023. Central section furthest from the basal part of the stem, length: 50 cm	Modena	44.592862, 10.994557
**L_1**	Hemp stem approximately 2 meters high harvested in 2020, of the Fedora variety. Basal section, length: 50 cm	Parma	44.837437, 10.434464
**L_2**	Hemp stem approximately 2 meters high harvested in 2020, of the Fedora variety. Central section closest to the basal part of the stem, length: 50 cm	Parma	44.837437, 10.434464
**L_3**	Hemp stem approximately 2 meters high harvested in 2020, of the Fedora variety. Central section furthest from the basal part of the stem, length: 50 cm	Parma	44.837437, 10.434464
**L_4**	Hemp stem approximately 2 meters high harvested in 2020, of the Fedora variety. Apical section of the stem, length: 50 cm	Parma	44.837437, 10.434464
**M**	Hemp presented in the form of short stems harvested in 2022	Brunico	46.803817, 11.927770
**N_1**	Hemp presented in the form of a short stem harvested in 2019	Modena	44.623222, 10.997917
**N_2**	Hemp presented in the form of short stems harvested in 2019	Modena	44.623222, 10.997917
**28**	Chopped hemp harvested in 2021, sample provided by CREA	Caserta	41.135526, 14.238501
**48**	Hemp harvested in 2022, sample provided by CREA	Bari	40.974270, 17.147109
**54**	Hemp harvested in 2022, sample provided by CREA	Rovigo	45.038195, 11.850637

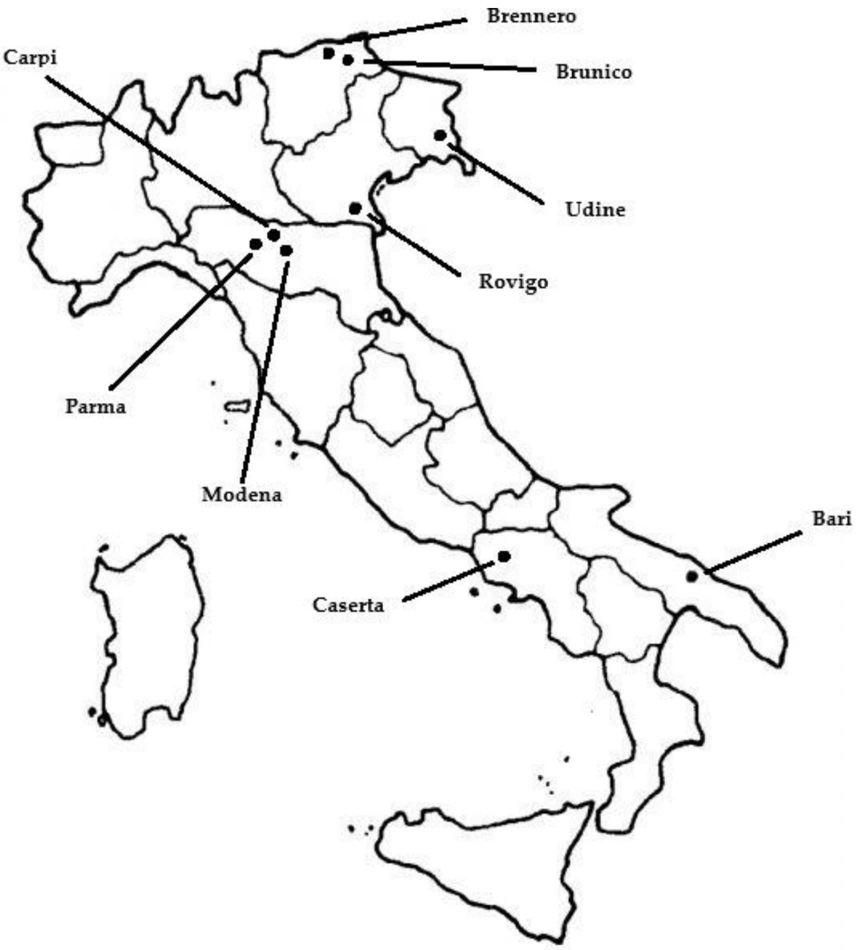

Figure S1. Map of sampling sites

Table S2. Mineralization ramp of the samples. E, microwave power; t, duration time of the step; T1, internal temperature of the container; T2, external limit temperature of the magnetron; P, maximum pressure of the mineralization chamber.

**Step**	**E** Watt	**t** min.	**T1** °C	**T2** °C	**P** **bar**
1	1500	5	100	60	160.0
2	1500	15	210	60	160.0
3	1500	5	210	60	160.0

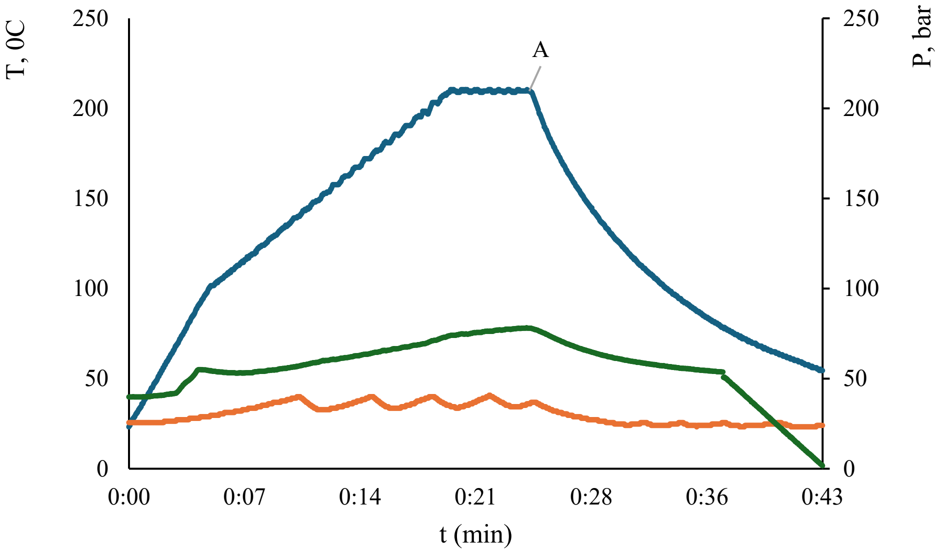

Figure S2. Trend of the temperature (blue line), pressure (green line) and autoclave cylinder temperature (orange line) parameters relating to the mineralization process of the hemp samples.

Table S3. Experimental conditions used for the determination of Li, Na, K, Mg and Ca using the FAAS technique.

**Element**	**Absorption/ Emission**	**Fiamma**	**Cs** ppm	**La** ppm	**λ** nm
**Li**	Emission	Acetylene/Air	2000	/	670.8
**Na**	Absorption	Acetylene/Air	2000	/	589.0
**K**	Absorption	Acetylene/Air	2000	/	766.5
**Mg**	Absorption	Acetylene/N_2O	/	5000	285.2
**Ca**	Absorption	Acetylene/Air	/	5000	422.7

Table S4. Heating program for the determination of Sr concentration using the GFAAS technique. The asterisked steps represent the signal reading stages.

Step	T<br>°C	t<br>s	Flow<br>L/min	Gas<br>Type
**1**	85	5.0	3.0	
**2**	95	40.0	3.0	
**3**	125	10.0	3.0	
**4**	210	5.0	3.0	
**5**	210	5.0	3.0	
**6**	1000	10.0	3.0	Ar
**7**	1000	8.0	3.0	
**8**	1000	2.0	0.0	
***9**	2700	0.9	0.0	
***10**	2700	2.5	0.0	
**11**	2700	5.0	3.0	

Table S5. Mass/charge ratio value measured for each element examined.

Element	Species measured	*m*/*z*
^{11}**B**	^{11}B	11
^{52}**Cr**	$^{52}Cr^{16}O$	68
^{55}**Mn**	^{55}Mn	55
^{56}**Fe**	^{56}Fe	56
^{59}**Co**	^{59}Co	59
^{60}**Ni**	^{60}Ni	60
^{63}**Cu**	^{63}Cu	63
^{66}**Zn**	^{66}Zn	66
^{85}**Rb**	^{85}Rb	85
^{88}**Sr**	$^{88}Sr^{16}O$	104
^{98}**Mo**	$^{98}Mo^{16}O$	114
^{111}**Cd**	^{111}Cd	111
^{118}**Sn**	^{118}Sn	118
^{138}**Ba**	$^{138}Ba^{16}O$	154
^{202}**Hg**	^{202}Hg	202
^{205}**Ti**	^{205}Ti	205
^{208}**Pb**	^{208}Pb	208
^{209}**Bi**	^{209}Bi	209

Table S6. Values of the elements measured for the control sample C.

Sample C				
**Element**	**Min**	**Max**	**Average**	**SD**
**Major component (mg/kg)**				
**K**	15402	15585	15473	98
**Ca**	11713	11913	11787	110
**Minor component (mg/kg)**				
**Mg**	1620	1656	1633	20
**Na**	547	633	589	43
**Fe**	471	506	486	11
**Trace component (mg/kg)**				
**Sr**	80	102	92	8
**Li**	55	66	61	6
**Mn**	30	33	31	1
**Zn**	26	30	27	1
**B**	20	22	21	1
**Ba**	17	20	18	1
**Cu**	6.58	7.59	7.07	0.33
**Rb**	3.94	4.30	4.10	0.12
**Ni**	1.62	1.95	1.73	0.11
**Cr**	1.58	1.77	1.67	0.06
**Pb**	0.59	0.70	0.64	0.03
**Sn**	0.21	0.26	0.24	0.02
**Co**	0.21	0.22	0.22	0.01
**Ultra-trace component (μg/kg)**				
**Mo**	81	93	86	4
**Cd**	57	62	60	1
**Bi**	8.38	9.64	8.89	0.41
**Tl**	7.60	8.93	8.20	0.42
**Hg**	1.54	1.99	1.77	0.16

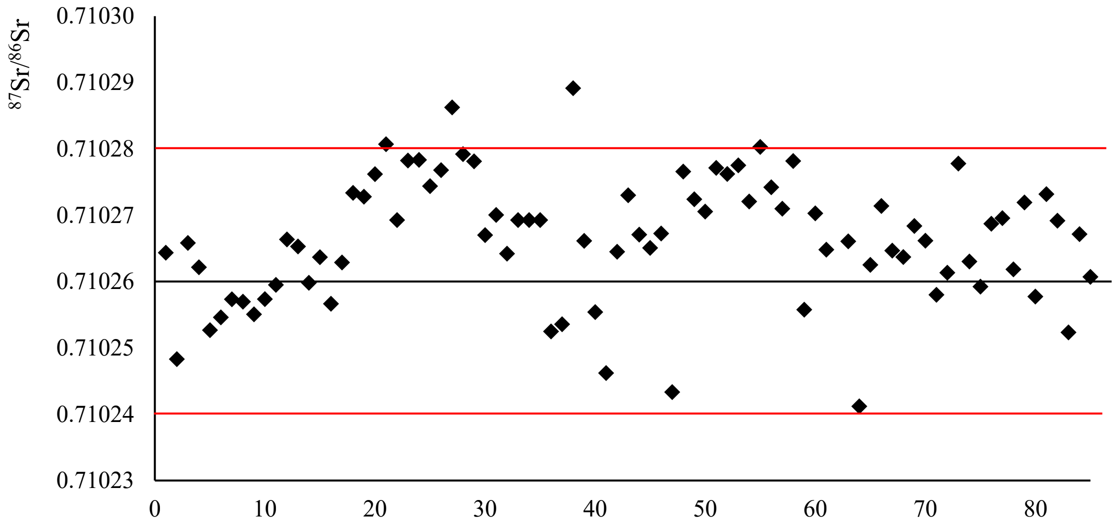

Figure S3. Trend of the isotope ratio values for the NIST 987 standard. The figure shows the generally accepted ratio value of 0.710263 and the associated uncertainty interval ±0.000016.

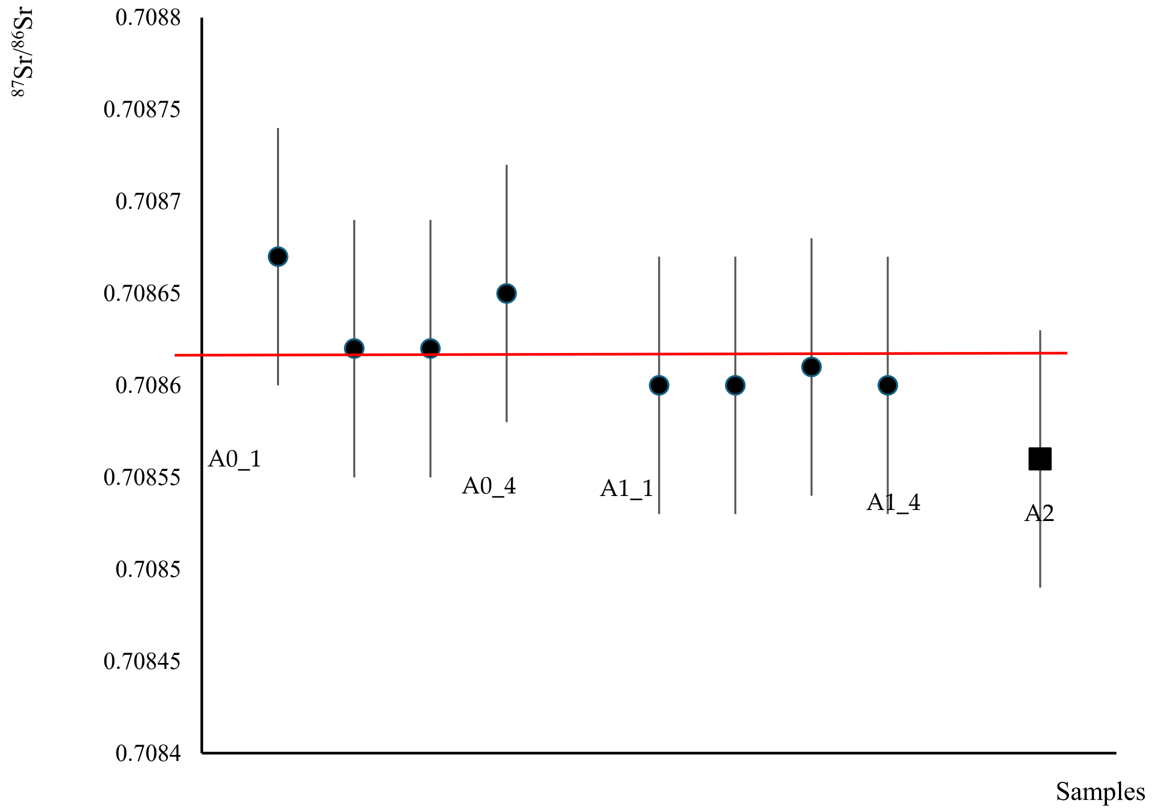

Figure S4. $^{87}Sr/^{86}Sr$ isotope ratio values determined on the hemp pieces (black circular indicator) and on the A2 fiber sample (square indicator). The vertical lines denote the uncertainty value, u = 2sd, associated with determinations equal to ±0.00005 for the series (A0_1-4 and A1_1-4) and ±0.00007 for the A2 fiber sample.

Table S7. Multi collector ICP-MS operating parameters for the $^{87}Sr/^{86}Sr$ ratio measurements.

**Parameter**	**Sr wet plasma**
**RF power (W)**	1245
**Gas flow rate (L min^{-1})**	Sample gas: 0.95 – 1.05<br>Auxiliary gas: 1.00<br>Cooling gas: 16
**Sample/Skimmer cone**	Ni
**Injector**	Sapphire
**Spray chamber**	Cyclonic + Scott type
**Nebulizer**	PFA micro flow self-aspirating
**Faraday Cup configuration - amplifiers**	L4 —<br>L3 – ^{83}Kr – 10^{12}<br>L2 – ^{84}Sr – 10^{12}<br>L1 – ^{85}Rb – 10^{12}<br>C – ^{86}Sr – 10^{11}<br>H1 – ^{87}Sr – 10^{11}<br>H2 – ^{88}Sr – 10^{11}<br>H3 —<br>H4 —
**Control Cup for peak center**	C – ^{86}Sr
**Mass analyzer pressure (mbar)**	$< 10^{-8}$
**Background/baseline determination**	4 % (v/v) HNO_3
**Sample uptake rate (µL min^{-1})**	100
**Uptake time (s)**	300
**Idle time (s)**	10
**Washing time (s)**	100
**Number of blocks**	1
**Number of cycles**	100
**Integration time (s)**	8.839
**Sensibility (V ppm^{-1})**	> 70 for ^{88}Sr
**Blank intensity range (mV)**	10 – 15 for ^{88}Sr
